# Gut Microbiota Variation With Short-Term Intake of Ginger Juice on Human Health

**DOI:** 10.3389/fmicb.2020.576061

**Published:** 2021-02-23

**Authors:** Xiaolong Wang, Dan Zhang, Haiqiang Jiang, Shuo Zhang, Xiaogang Pang, Shijie Gao, Huimin Zhang, Shanyu Zhang, Qiuyue Xiao, Liyuan Chen, Shengqi Wang, Dongmei Qi, Yunlun Li

**Affiliations:** ^1^Experimental Center, Shandong University of Traditional Chinese Medicine, Jinan, China; ^2^Key Laboratory of Traditional Chinese Medicine Classical Theory, Ministry of Education, Shandong University of Traditional Chinese Medicine, Jinan, China; ^3^Shandong Provincial Key Laboratory of Traditional Chinese Medicine for Basic Research, Shandong University of Traditional Chinese Medicine, Jinan, China; ^4^Beijing Institute of Radiation Medicine, Beijing, China; ^5^Affiliated Hospital of Shandong University of Traditional Chinese Medicine, Jinan, China

**Keywords:** ginger juice, human gut microbiota, prevotella-to-bacteroides ratio, firmicutes-to-bacteroidetes ratio, *Faecalibacterium*

## Abstract

Ginger, a widely used functional food and food additive, little is known about the effect of ginger juice, which is rich in many healthful agents, on healthy humans or on its relationship with gut microbiota composition variation. The aim of this study was to investigate the changes in the gut microbial communities that occur following the supplementation of fresh ginger-derived juice in healthy adults and its potential associations with function. A crossover intervention study in which 123 healthy subjects (63 men and 60 women) consumed fresh ginger juice from *Zingiber officinale Rosc*. or sterile 0.9% sodium chloride was conducted. 16S rRNA sequencing analyses were applied to characterize gut microbiota variation. We found that ginger juice intervention increased the species number of intestinal flora. A decreased relative abundance of the Prevotella-to-Bacteroides ratio and pro-inflammatory *Ruminococcus_1* and *Ruminococcus_2* while a tendency toward an increased Firmicutes-to-Bacteroidetes ratio, Proteobacteria and anti-inflammatory *Faecalibacterium* were found. When we did not consider gender, we found differences in bacterial diversity both in community evenness and in richness caused by ginger intervention. In fact, there were different changes in bacterial α-diversity induced by the ginger juice in men and women. We identified 19 bacterial genera with significant differences between the control group (women) and ginger group (women) and 15 significant differences between the control group (men) and ginger group (men) at the genus level. Our results showed that short-term intake of ginger juice had substantial effects on the composition and function of gut microbiota in healthy people. Moreover, our findings underscored the importance of analyzing both male and female individuals to investigate the effects of ginger on gut microbiota. Additional studies are necessary to confirm these findings.

## Introduction

Ginger (*Zingiber officinale*) has longstanding historical medicinal use. In the present day, from juices of the fresh rhizome, to ginger powder and ginger essential oil is growing in popularity for universal health benefits. The health-promoting perspectives of ginger are well documented and the FDA ranked it in the generally recognized as safe (GRAS) list. The use of ginger as a dietary supplement and functional food is a growing trend (Krell and Stebbing, 2012; Yilmaz et al., 2018; Jiang, 2019).

Over 60 active constituents are present in ginger, which can be broadly divided into volatile components (mainly composed of hydrocarbons) and nonvolatile pungent components, such as gingerols, paradols, shogaols, and zingerone (Choi, 2019). Its pharmacological properties are varied, including antioxidant and anti-inflammatory abilities (Jiang et al., 2013; Mozaffari-Khosravi et al., 2016), antiplatelet and hypolipidemic abilities (Alizadeh-Navaei et al., 2008; Nicoll and Henein, 2009), and antiglycation and antiglycemic effects (Arablou et al., 2014; Shidfar et al., 2015). In addition, it also has been used as a remedy for nausea and vomiting (Dabaghzadeh et al., 2014; Bameshki et al., 2018), cardiovascular health (Han et al., 2008), and joint and muscle health (Matsumura et al., 2015), and it also has the potential to aid in managing weight (Miyamoto et al., 2015).

Clinical trials show ginger (1 g/day) may be safe and effective for decreasing nausea and vomiting during pregnancy or for nausea induced by chemotherapy (Pongrojpaw et al., 2007; Levine et al., 2008; Ozgoli et al., 2009). A double-blind, placebo-controlled trial with 85 hyperlipidimic subjects showed that 3 g/day ginger for 45 days markedly lowered blood levels of triglyceride (TG), cholesterol (CHOL), and low-density lipoprotein (LDL), with increased high-density lipoprotein (HDL), when compared with a placebo control (Alizadeh-Navaei et al., 2008). It was also reported that 4 g ginger supplementation could accelerate muscle strength recovery following intense exercise in a randomized trial on 20 nonweight-trained participants (Matsumura et al., 2015). Ginger consumption (2 g/day ginger powder) for 12 weeks in women with obesity showed a significant decrease in body mass index (BMI), serum insulin, and homeostatic model assessment-insulin resistance index, and total appetite score (Miyamoto et al., 2015).

The gut microbiota is a complex microbial community that interacts with one another and with the host organism, influencing many aspects of human health (Jackson et al., 2018; Durack and Lynch, 2019). Most ingested compounds, whether taken for dietary, therapeutic benefits, or other purposes, influence the microbiota, and conversely the microbiota also can metabolize many orally ingested substances (Hansen et al., 2018; Riccio and Rossano, 2018). Ginger supplementation can modulate the composition of the gut microbiota, resulting in its effects on obesity, insulin resistance, liver steatosis, and low-grade inflammation in mice (Wang et al., 2019). Gingerols and shogaols in ginger are rapidly absorbed in the small intestine and have been shown to be metabolized by human gut microbiota. Preliminary data also have indicated potential gut microbiome modulatory effects (Wang et al., 2017; Thumann et al., 2019). Recent studies have shown that ginger-derived exosomes-like nanoparticles (GELNs) are taken up by the gut microbiota and contain RNAs that alter microbiome composition and host physiology. These functions of GELN RNAs can ameliorate mouse colitis through IL-22-dependent mechanisms (Teng et al., 2018).

Many clinical trials have confirmed the effect of ginger supplementation on the regulation of specific human health. Additionally, most of the studies from animal experiments have focused on a single bioactive ingredient and the influence of herbal extracts containing ginger on selected bacterial populations. Ginger juice is convenient to obtain and is rich in many healthful agents; however, no reports have examined the impact of ginger juice, as a supplementary alternative treatment, on the human gut microbiota composition. Thus, we set out to rectify this lack in research by analyzing fecal material collected throughout a fresh ginger juice supplementary intervention study.

**Figure 1:**
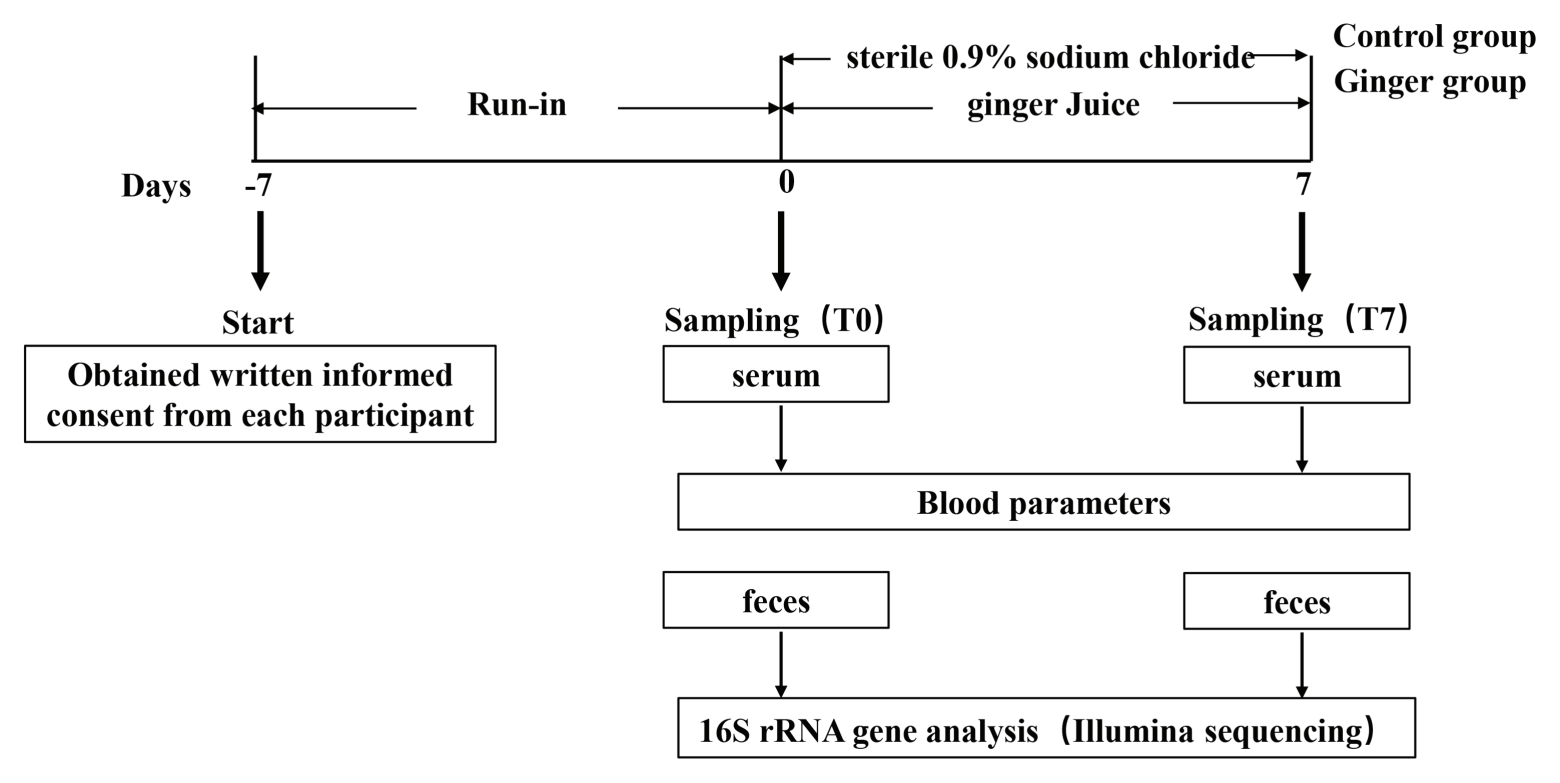
Schematic view of the study design. This study included 123 participants and was divided in two periods of 1 week each, as detailed in the Materials and Methods section. Blood samples were collected to measure blood biochemical items, while fecal samples were collected to analyze gut microbiota variation by 16S rRNA gene sequencing, respectively, at time points T0 and T7 of the study.

We set up this trial as a randomized and controlled trial during which 123 healthy adults consumed fresh ginger-derived juice or sterile 0.9% sodium chloride on a daily basis for a 1-week intervention period and a 1-week run-in period before consecutive interventions (Figure 1). We analyzed the samples collected to assess the impact of ginger juice intervention on fecal microbiota composition profiles using 16S rRNA gene amplicon sequencing.

## Materials and Methods

### Study Participants

We recruited 123 young (20–30 years; 63 men and 60 women) healthy individuals (BMI, 18.5–23.9 kg/m^2^) of Chinese ancestry from Shandong University of Traditional Chinese Medicine. We excluded individuals with a self-reported history of allergy to ginger; bowel disorders, such as irritable bowel syndrome or inflammatory bowel disease; chronic diseases, such as allergies, diabetes, or hemorrhoids; and antibiotic, prebiotic, or probiotic treatment within 2 months preceding the study.

The study took place at the experimental center of Shandong University of Traditional Chinese Medicine, China. We conducted this research in compliance with the Helsinki Declaration, and the study was approved by the Ethics Committee of Affiliated Hospital of Shandong University of Traditional Chinese Medicine (Approved No. of ethic committee: 2020-004-KY). We obtained written informed consent from each participant. The clinical aspects of the study were supervised by a physician. A code was assigned to each participant by the study coordinator at inclusion to ensure blinded handling of samples by the technical staff.

### Study Design and Sample Collection

We set up this study as a randomized and controlled trial targeted to investigate the effect of ginger juice on gut microbial patterns in healthy individuals. This study consisted of two main periods of 1 week each (Figure 1): (1) a run-in period, in which participants consumed their usual diet but were asked not to consume the ginger-rich products, as well as not to take pro‐ and pre-biotics; and (2) an intervention period, during which either ginger juice (ginger group) or sterile 0.9% sodium chloride (control group) was consumed on a daily basis. A sample size of at least 60 participants per group, which is half men and half women. The participants were instructed to drink 20 ml from 8:00 to 8:30 am every day at the experimental center of the university campus where the ginger juice was freshly squeezed.

At the start of the study and on two occasions thereafter (time points T0–T7), we monitored the participants’ medical status with the help of questionnaires. Blood samples were drawn *via* an intravenous cannula in the participants’ antecubital vein in the specialized outpatient clinic at the affiliated hospital. Shortly after collection, the blood samples were stored at room temperature for 30 min, separated into serum and plasma, and obtained serum by centrifugation (15 min, 2,000 × *g*) at 4°C. Serum alanine transaminase (ALT), aspartate aminotransferase (AST), cholinesterase (CHE), creatinine (Cr), TG, CHOL, high-density lipoprotein cholesterol (HDL-C), low-density lipoprotein cholesterol (LDL-C), glucose (GLU), and creatine kinase isoenzymes (CK-MB) were analyzed using automated, enzymatic, colorimetric assay on URIT biochemical analyzer (URIT-8020, URIT Medical Electronic Co., Ltd. Guilin, China). We analyzed the serum samples at T0 taken at the start of the study to determine basal participants’ characteristics and to eliminate the possibility of illness. We also took serum samples at T7 for biochemical items analyses to follow the possible effects of the ginger intervention.

Participants were provided waxed tissue paper, which was laid down on the water in the toilet bowl prior to defecation. This paper sticks to the sides of the toilet bowl so that the fecal sample is not readily contaminated by water in the toilet. Fresh fecal samples (around 1 g) were collected at sampling point T0 and T7, suspended in stool storage solution according to the manufacturer’s protocol of Longseegen stool storage kit (LS-R-P-003, Longsee Biomedical Corporation, Guangzhou, China). The kits were returned within 24 h of collection. The Longseegen stool storage tube contains 2 ml of a stabilization buffer, which can slow down the interference of microbial fermentation on the sample and ensure the freshness of the sample to a certain extent. All of the fecal samples were divided into two equal parts after delivery to the laboratory staff and were stored at −80°C until further processing.

### Preparation of Ginger Juice and Dosage Information

We purchased fresh *Zingiber officinale Roscoe* from Laiwu, Shandong Province, People’s Republic of China. We washed, dried, and preserved the ginger at 4°C. Briefly, we prepared the ginger juice daily using a juice extractor, which directly separated the dregs and the juice before the subjects came to the experimental center to start the test. The laboratory staff removed water-insoluble materials and retained only the juice. Finally, ginger juice with a concentration of 1.5 g/ml was prepared at room temperature. The ginger group received daily 20 ml of fresh ginger juice (If a person’s weight is calculated as 60 kg, it is equivalent to 500 mg/kg/day) orally for 7 days. Participants in the control group received an equal volume of sterile 0.9% sodium chloride without the added ginger juice, also by oral administration.

### DNA Extraction and PCR Amplification

Microbial DNA was extracted from 246 samples using the E.Z.N.A.® soil DNA Kit (Omega Bio-tek, Norcross, GA, United States) according to manufacturer’s protocols. The final DNA concentration and purification were determined by NanoDrop 2000 UV-vis spectrophotometer (Thermo Scientific, Wilmington, United States), and DNA quality was checked by 1% agarose gel electrophoresis. The V3-V4 hypervariable regions of the bacteria 16S rRNA gene were amplified with primers 338F (5'-ACTCCTACGGGAGGCAGCAG-3') and 806R (5'-GGACTACHVGGGTWTCTAAT-3') by thermocycler PCR system (GeneAmp 9,700, ABI, United States). The PCR reactions were conducted using the following program: 3 min of denaturation at 95°C, 27 cycles of 30 s at 95°C, 30s for annealing at 55°C, and 45 s for elongation at 72°C, and a final extension at 72°C for 10 min. PCR reactions were performed in triplicate 20 μl mixture containing 4 μl of 5 × FastPfu Buffer, 2 μl of 2.5 mM dNTPs, 0.8 μl of each primer (5 μM), 0.4 μl of FastPfu Polymerase and 10 ng of template DNA. The resulted PCR products were extracted from a 2% agarose gel and further purified using the AxyPrep DNA Gel Extraction Kit (Axygen Biosciences, Union City, CA, United States) and quantified using QuantiFluor™-ST (Promega, United States) according to the manufacturer’s protocol.

### Illumina Miseq Sequencing

Purified amplicons were pooled in equimolar and paired-end sequenced (2 × 300) on an Illumina MiSeq platform (Illumina, San Diego, United States) according to the standard protocols.

### Bioinformatics Analysis

The analysis was conducted by following the “Atacama soil microbiome tutorial” of Qiime2docs along with customized program scripts.^1^ Briefly, raw data FASTQ files were imported into the format, which could be operated by QIIME2 system using qiime tools import program. Demultiplexed sequences from each sample were quality filtered and trimmed, de-noised, merged, and then the chimeric sequences were identified and removed using the QIIME2 dada2 plugin to obtain the feature table of amplicon sequence variant (ASV; Callahan et al., 2016). The QIIME2 feature-classifier plugin was then used to align ASV sequences to a pre-trained GREENGENES 13_8 99% database (trimmed to the V3V4 region bound by the 338F/806R primer pair) to generate the taxonomy table (Bokulich et al., 2018). Then, we analyzed the available data on the free online platform of Majorbio I-Sanger Cloud Platform (www.i-sanger.com, Majorbio, China).

We implemented a Venn diagram using the R package to show unique and shared operational taxonomic units (OTUs). We calculated alpha-diversity analyses, including microbial community richness parameters (Chao1), and community diversity parameters (Shannon) using the mothur software. We assessed differences among treatment groups for α-diversity according to a Kruskal-Wallis multiple comparison followed by Wilcoxon pairwise comparison for significantly different groups. Beta diversity distance measurements (Bray Curtis) were performed to investigate the structural variation of microbial communities across samples and then visualized *via* principal coordinate analysis (PCoA). We used analysis of similarities (ANOSIM) and a permutational multivariate ANOVA (PERMANOVA) test for β-diversity to determine microbial composition and structure differences between groups, followed by a pairwise PERMANOVA. We generated a heatmap on the basis of the relative abundance of the phyla using R software. In addition, the potential Kyoto Encyclopedia of Genes and Genomes (KEGG) Ortholog (KO) functional profiles of microbial communities were predicted with PICRUSt (Langille et al., 2013).

## Results

### Baseline Characteristics of Study Participants

**Figure 2:**
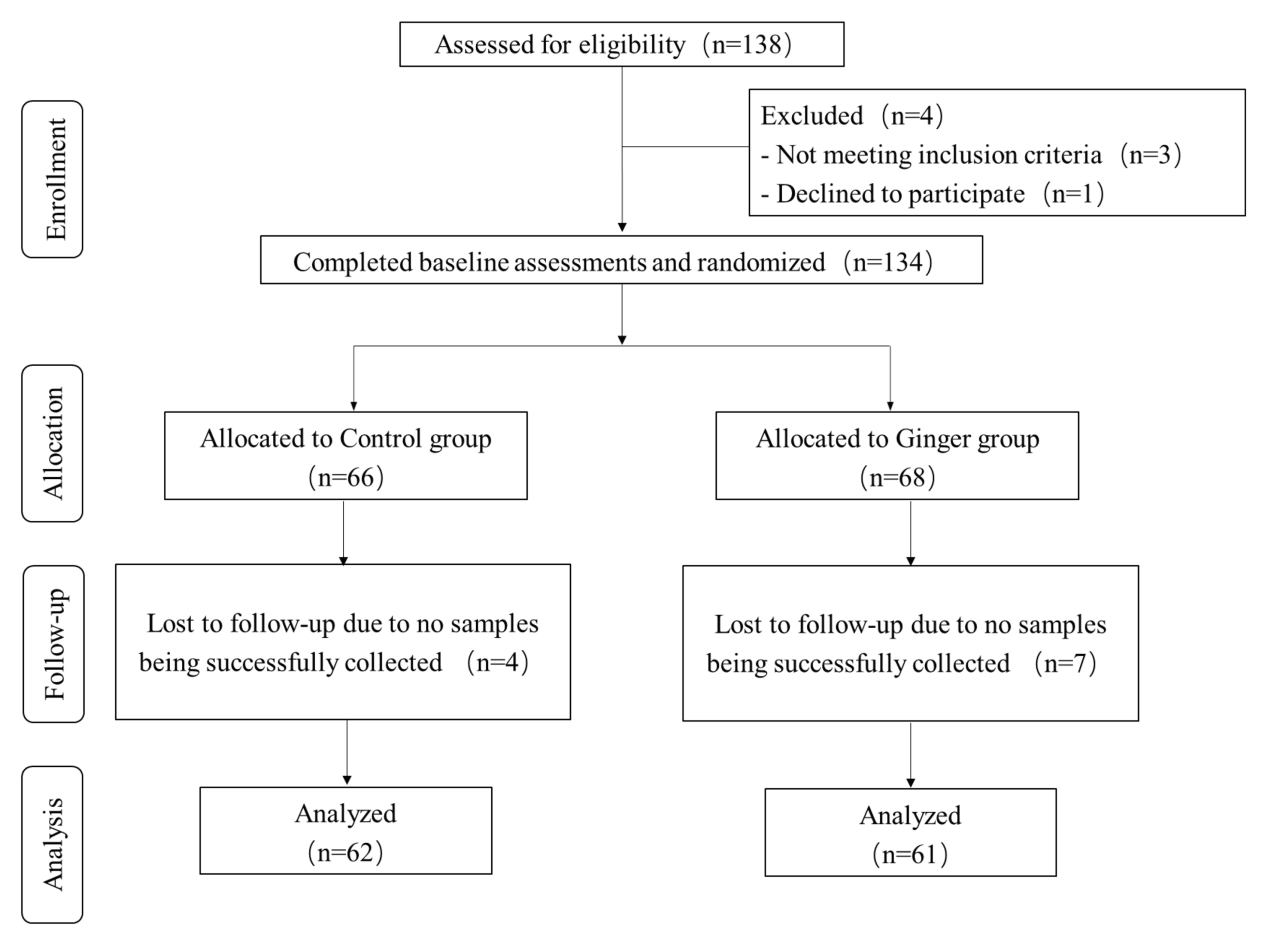
Flow diagram of the healthy subjects.

We assessed a total of 138 individuals for eligibility of which three did not meet the inclusion criteria and one declined to participate. During the intervention phase of the study, we excluded four people from the control group [no samples were successfully collected for personal reasons (*n* = 4)] and seven people from the intervention group [no samples were successfully collected for personal reasons (*n* = 7)]. We included 123 healthy volunteers (63 men and 60 women) who were included in the final analysis (Figure 2). On average, the rate of compliance in our study was high. We did not observe any significant differences in anthropometric measurements or clinical parameters, including the mean age, weight, and BMI, among the ginger-supplement and control groups at baseline (T0). Twelve blood biochemical indexes were within the normal range, and the proportion of men and women in each group was average (Table 1). After intervention, we selected 246 fecal samples (T0 and T7) for 16S rRNA gene sequence analysis.

**Table 1:** Baseline characteristics of the participants included in the study (*n* = 123).

Characteristic	Control group (*n* = 62)	Ginger group (*n* = 61)	Medical reference range of blood parameters
Age (years)	21.10 (1.95)	20.50 (0.84)	–
Men	32 (26.0%)	31 (25.2%)	–
Women	30 (24.4%)	30 (24.4%)	–
Weight (kg)	61.80 (10.83)	65.09 (13.64)	–
BMI (kg/m^2^)	21.07 (2.31)	21.69 (3.86)	–
ALT (U/L)	15.81 (7.72)	18.07 (11.15)	0–40
AST (U/L)	19.71 (3.55)	21.42 (4.97)	0–40
ALT/AST	0.78 (0.30)	0.81 (0.36)	1.0–1.2
CHE (U/L)	9924.52 (2022.62)	10564.66 (1853.12)	3,700–13,200
Urea (mmol/L)	5.22 (1.11)	4.83 (1.02)	2.50–8.20
Cr (mmol/L)	75.23 (12.27)	75.54 (13.05)	53–123
TG (mmol/L)	0.96 (0.50)	1.04 (0.43)	0.00–1.71
CHOL (mmol/L)	4.29 (0.92)	4.30 (0.81)	3.60–6.50
HDL-C (mmol/L)	1.46 (0.25)	1.38 (0.24)	0.83–1.96
LDL-C (mmol/L)	2.19 (0.65)	2.32 (0.62)	2.06–3.10
GLU (mmol/L)	5.16 (0.40)	5.13 (0.35)	3.90–6.10
CK-MB (U/L)	5.58 (5.34)	6.10 (3.65)	0–25

Data are mean (SD) or n (%). BMI, body mass index; ALT, alanine transaminase; AST, aspartate aminotransferase; CHE, cholinesterase; Cr, serum creatinine; TG, triglyceride; CHOL, cholesterol; HDL-C, high-density lipoprotein cholesterol; LDL-C, low-density lipoprotein cholesterol; GLU, glucose; and CK-MB, creatine kinase isoenzymes.

### Ginger Intervention Increased the Species Number of Intestinal Flora

**Figure 3:**
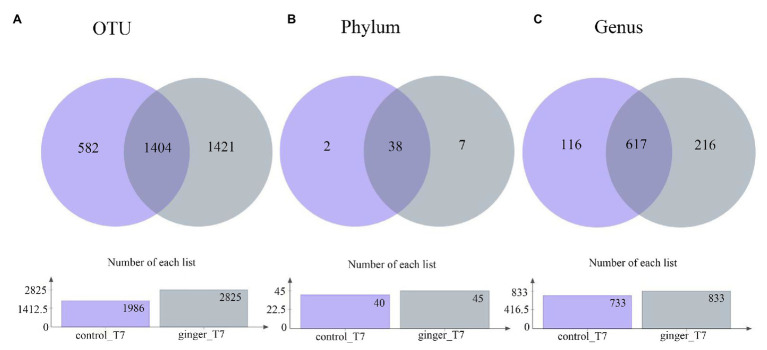
Number of taxonomic composition. The taxonomic composition after intervention (time point T7) in each group at **(A)** operational taxonomic unit (OTU) level, **(B)** phylum level, and **(C)** genus level. In these figures, different colors represent different groups, the number of overlapping parts represents the number of species shared by the control group and ginger group, and the number of nonoverlapping parts represents the number of species unique to the corresponding group. The lower figures show the total number of species in each group at different taxonomic levels.

Across the 123 samples (control group and ginger group at time point T7), we assigned gene sequences to 3,407 OTUs in total. We distributed the detected OTUs among the 47 different bacterial phyla and 949 different bacterial genera in total at the phylum level and genus level, respectively (Figure 3). The 38 phyla were shared by the two groups and the seven phylum-level intestinal flora was unique to the ginger group, including the Peregrinibacteria phylum, Omnitrophica phylum, and Berkelbacteria phylum. After ginger intervention, the species composition increased significantly at multiple taxonomic levels.

**Figure 4:**
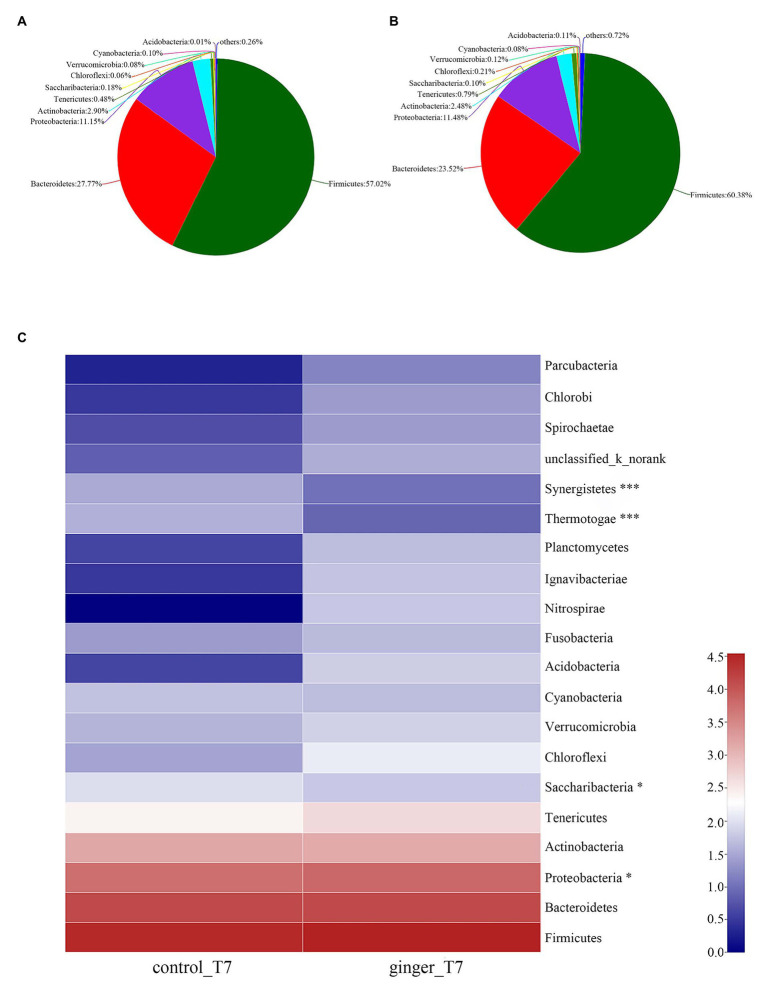
Gut microbiota composition changes between the control and ginger groups at time point T7. Relative abundance of bacterial groups in the feces of the **(A)** control group samples and the **(B)** ginger group samples at the phylum levels. Less than 0.1% abundance of the phyla was merged into others. **(C)** Community heatmap analysis. The abscissa is the group name, and the ordinate is the bacterial species name at the phylum levels. The intensity of the colors represented the degree of the abundance of different species in the two groups of samples. Significant species are marked by ^*^p < 0.05; ^***^p < 0.001.

As Figures 4A,B shown that, we attributed most of the OTU sequences to 10 phyla: Firmicutes, Bacteroidetes, Proteobacteria, Actinobacteria, Tenericutes, Saccharilbacteria, Cyanobacteria, Verrucomicrobia, Chloroflexi, and Acidobacteria. The most abundant phyla in all of the volunteers were Firmicutes, Bacteroidetes, and Proteobacteria. The control group was characterized by Firmicutes (57.02%), Bacteroidetes (27.77%), and Proteobacteria (11.15%), whereas the ginger group showed Firmicutes (60.38%), Bacteroidetes (23.52%), and Proteobacteria (11.48%) levels. The ginger group presented the higher Firmicutes and Proteobacteria and a lower Bacteroidetes level. Ginger supplementation increased the Firmicutes-to-Bacteroidetes ratio and Proteobacteria abundance at time point T7.

To evaluate the significant differences between the control group and ginger group, we compared the two groups at the two time point of the intervention period, respectively. All of the observed variations at the phylum level did not attain the statistical significance at time point T0 (not shown). At time point T7, the ginger intervention-induced difference in microbiota composition is illustrated in Figure 4C. Compared with the control group, the relative abundance of Proteobacteria significantly increased and Saccharibacteria, Thermotogae, and Synergistetes significantly decreased in ginger (corrected value of *p* = 0.046, 0.048, 1.812E-11, and 2.269E-8, respectively).

### Different Changes in Fecal Microbiota With Ginger Intervention in Men and Women

**Figure 5:**
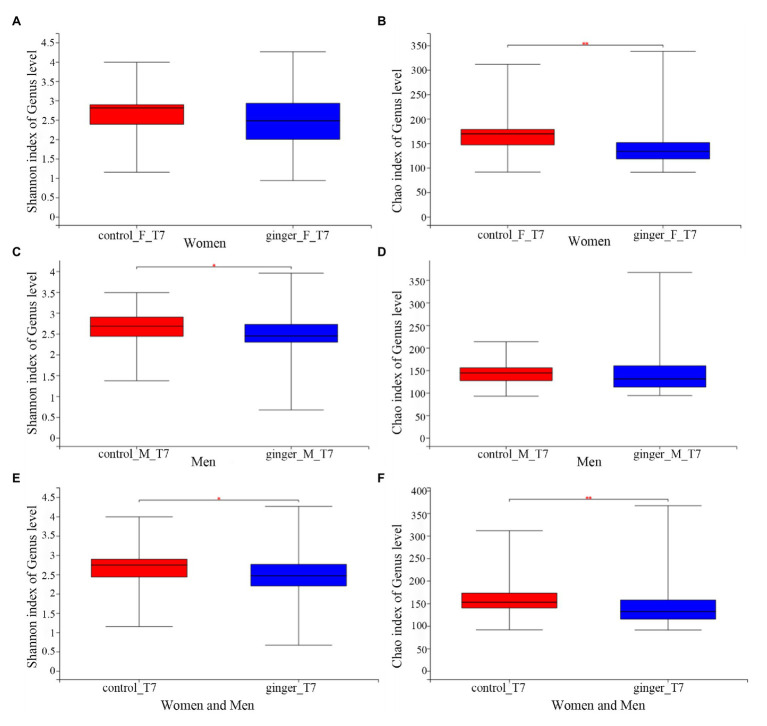
The effect of intervention of ginger juice on fecal microbiota based on alpha diversity indices. Alpha diversity was evaluated by **(A)** Shannon index, **(B)** Chao1 index at time point T7 between the control and ginger groups in the female subjects. Alpha diversity was evaluated by **(C)** Shannon index, **(D)** Chao1 index at time point T7 between the control and ginger groups in the male subjects. Alpha diversity was evaluated by **(E)** Shannon index, **(F)** Chao1 index at time point T7 between the control and ginger groups including all of the female and male subjects. We used the Wilcoxon rank-sum test to highlight significant differences, ^*^p < 0.05; ^**^p < 0.01.

We assessed the effect of intervention of ginger juice on fecal microbiota based on α-diversity indices and β-diversity. We used the Wilcoxon rank-sum test to highlight significant differences in the α-diversity term for the Shannon index and Chao1 index for all of the control and ginger groups at time points T0 and T7.α-Shannon (not shown, value of *p* = 0.36) and Chao1 (not shown, value of *p* = 0.31) diversity was not different between the two groups at time point T0. As shown in Figure 5, we observed significant differences (*p* < 0.05) in the α-Shannon index and Chao’s index at time point T7 between the control and ginger groups (including all of the female and male subjects, Figures 5E,F). Thus, we observed significant changes in microbial community evenness and in richness for those participants who received a daily supplement of ginger juice compared with the sterile 0.9% sodium chloride supplement. Note that changes in bacterial α-diversity induced by the ginger intervention were different in the female and the male subjects. At time point T7, adding ginger juice daily significantly decreased women’s Chao1 diversity (Figure 5B); however, Shannon’s indices (Figure 5A) were not different between the control and ginger groups. Conversely, in the male samples, we found significant differences in the Shannon index (Figure 5C) between the two groups, but there was no difference in the Chao1 index (Figure 5D). This means that we found significant changes only in microbial community evenness (Shannon’s index) for men, while we found a significant change only in richness (Chao1) for women. When we did not consider gender, we found differences in bacterial diversity both in community evenness and in richness caused by ginger intervention.

**Figure 6:**
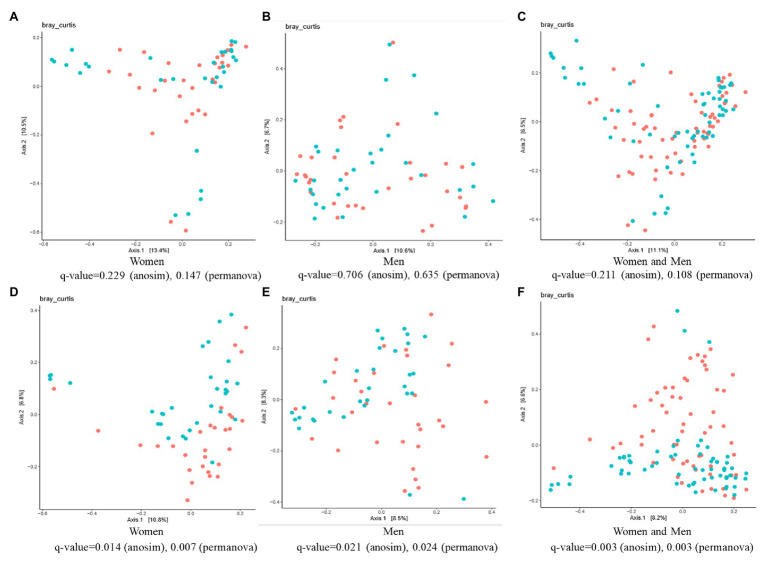
The effect of intervention of ginger juice on fecal microbiota based on β-diversity. Principal coordinate analysis (PCoA) plots of fecal microbiotas were generated of the control group (red circle) and ginger group (green circle) samples at **(A–C)** time point T0 [for women **(A)**, for men **(B)**, for both women and men **(C)**] and **(D–F)** T7 [for women **(D)**, for men **(E)**, for both women and men **(F)**]. Analysis of similarities (ANOSIM) and permutational multivariate ANOVA (PERMANOVA) tests demonstrate that ginger supplementation significantly altered the composition of microbiota in most individuals in the study.

The β-diversity analysis highlighted the existence of a significant difference between the control and ginger groups for both female and male subjects, although we did not detect any differences (value of *q* > 0.05) among the samples at time point T0 (Figures 6A–C). At time point T7 (Figures 6D–F), ANOSIM tests showed that ginger supplementation altered the composition of microbiota in most individuals in the study (value of *q* = 0.003 for both women and men; value of *q* = 0.014 for women; value of *q* = 0.021 for men, respectively). This conclusion was corroborated by results from a PERMANOVA to determine whether ginger supplementation altered the composition of microbiota in the overall study population. To incorporate intraindividual variability, each individual was considered as a blocking factor in the PERMANOVA. This analysis revealed that the composition of the gut microbiota was altered with ginger supplementation in our study population (PERMANOVA using Bray-Curtis similarity blocking for each individual; value of *q* = 0.003 for both women and men; value of *q* = 0.007 for women; value of *q* = 0.024 for men, respectively).

### Unique and Shared Bacterial Taxa at the Phylum and Genus Levels Induced by Ginger Between Individual Women and Men

**Figure 7:**
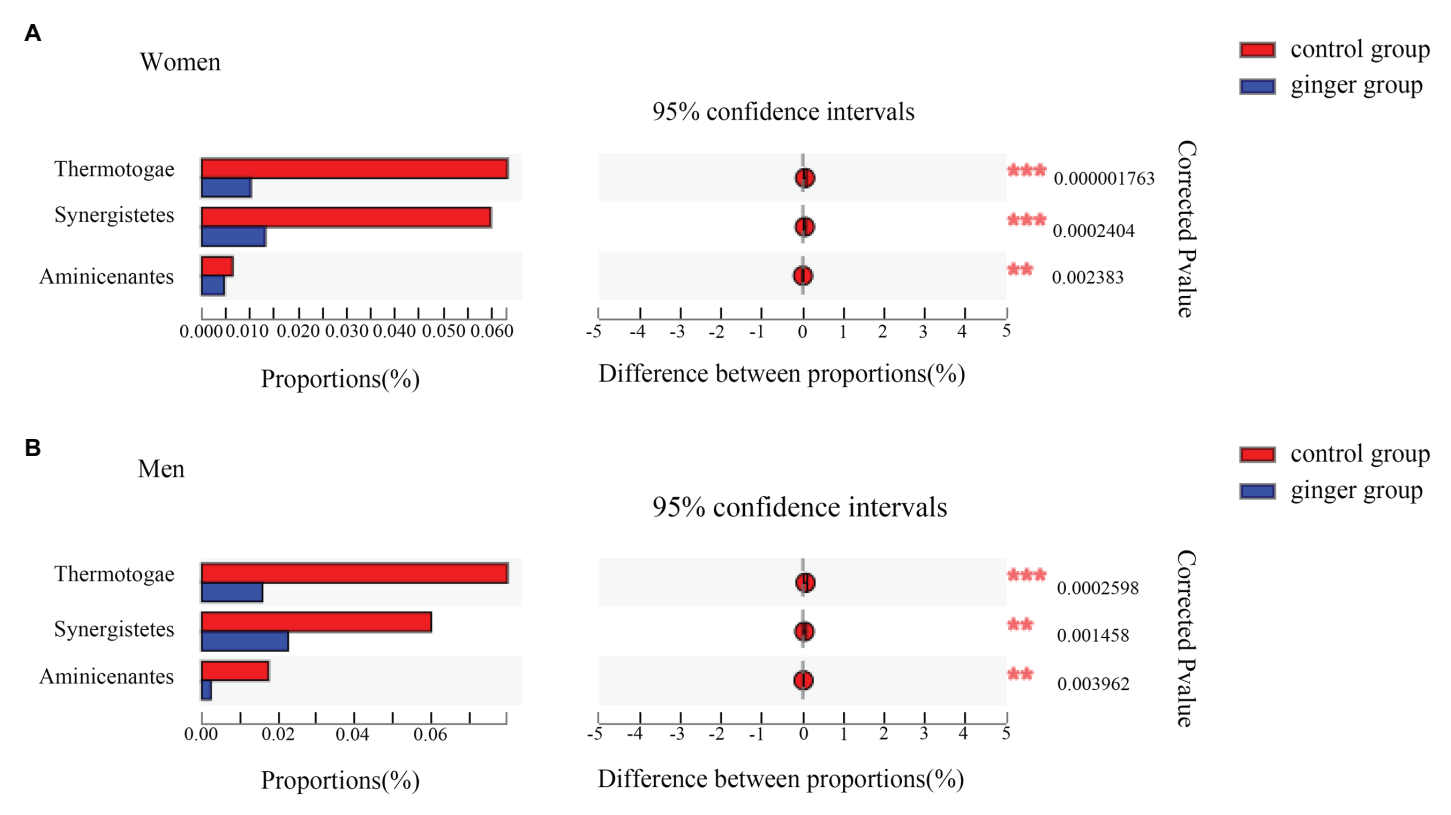
Relative abundance of significantly different phyla between the control and ginger groups. Bar plots of Wilcoxon rank-sum test at the phylum level for **(A)** women and for **(B)** men. Corrected value of *p* are shown at right.

Next, based on the community abundance data in the samples, we detected the bacterial species with different abundances in different groups of microbial communities. We carried out a Wilcoxon rank-sum test to evaluate the significance of the observed differences. As shown in Figure 7, regardless of whether the subject was male or female, we observed three statistically significant differences among the bacterial phylum between the control and ginger groups at the phyla level, including Thermotogae, Synergistetes, and Aminicenantes. The relative abundance of these phylum was significantly lower in the ginger group compared with the control group.

**Figure 8:**
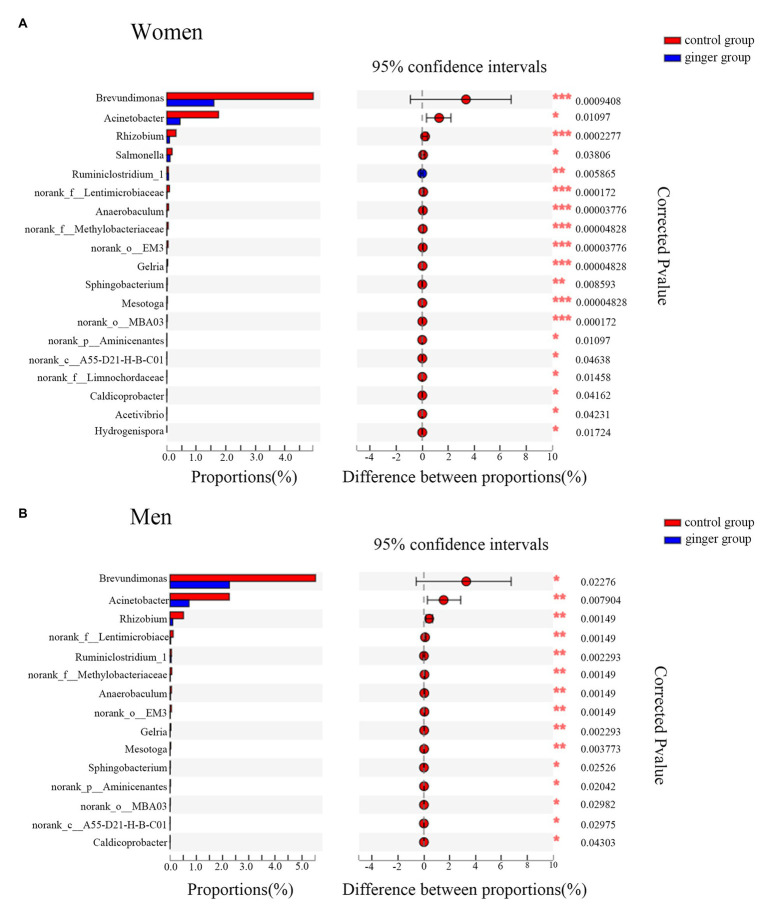
Relative abundance of significantly different genera between the control and ginger groups. Bar plots of Wilcoxon rank-sum test at the genus level for **(A)** women and for **(B)** men. Corrected value of *p* are shown at right.

A Wilcoxon rank-sum test bar plot for the genus level is shown in Figure 8. We identified 19 statistically significant differences between the control group (women) and ginger group (women) and identified 15 significant differences between the control group (men) and ginger group (men) at the genus level. Although 15 bacterial genera with significant differences between the control group and ginger group, including *Rhizobium*, *norank_f__Lentimicrobiaceae*, *norank_f__Methylobacteriaceae*, *Anaerobaculum*, *norank_o__EM3*, *Ruminiclostridium_1*, *Gelria*, *Mesotoga*, *Acinetobacter*, *g__norank_p__Aminicenantes*, *Brevundimonas*, *Sphingobacterium*, *norank_c__A55-D21-H-B-C01*, *norank_o__MBA03*, and *Caldicoprobacter* were shared by the female and male subjects, their relative abundance was differentially represented. For example, for women, the relative abundance of *Brevundimonas* (1.595 vs. 4.958%; corrected value of *p* < 0.001), *Acinetobacter* (0.449 vs. 1.75%; corrected value of *p* = 0.01), and *Rhizobium* (0.09 vs. 0.312%; corrected value of *p* < 0.001), whereas for men, the relative abundance of *Brevundimonas* (2.255 vs. 5.53%; corrected value of *p* < 0.001), *Acinetobacter* (0.722 vs. 2.25%; corrected value of *p* = 0.01), *Rhizobium* (0.101 vs. 0.513%; corrected value of *p* < 0.001) were all significantly lower in the ginger group compared with the control group. For women, there were four unique differentially expressed genera, including *Hydrogenispora*, *Salmonella*, *Acetivibrio*, and *norank_f__Limnochordaceae* were observed.

### Predicted Metagenome Inference

We used the PICRUSt software package^2^ to predict the functional composition of a microbial community’s metagenome from its 16S profile. The value of PICRUSt depended on the accuracy of its predicted metagenomes from marker gene samples and the corresponding ability to recapitulate findings from metagenomic studies. Because 16S rRNA copy number varied greatly among different bacteria and archaea, we normalized our table of OTUs by dividing the abundance of each organism by its predicted 16S copy number. We then multiplied the normalized OTU abundances by the set of gene family abundances calculated for each taxon during the gene content inference step. Then, we conducted a functional annotation of KEGG of OTU according to the green ID corresponding to each OTU and obtained the annotation information of OTU at each functional level KEGG and the abundance information of each function for the different samples.

After completing the KEGG function annotation, combined with group information, we used ANOVA tests to analyze whether there was significant difference in microbial community prediction function between groups. Before the ANOVA test, we first screened out the metabolic pathways (filter the pathways observed in less than 50% of total samples) and then analyzed the differences based on relative abundance. Table 2 shows the pathways (L1–L3 levels) with all of the significant differences obtained by ANOVA tests between the control and ginger group at time point T7. Twelve pathways were involved in human diseases, and among them, two were pathways for cardiovascular diseases, namely, viral myocarditis and hypertrophic cardiomyopathy; three pathways involved immune system diseases; four were infectious disease pathways; and three pathways were cancer-related pathways. Fourteen pathways focused on metabolism. One of the metabolism pathways involved lipid metabolism and two of these pathways were metabolism of amino acids. They also included biosynthesis of other secondary metabolites, such as flavonoid and indole alkaloid biosynthesis pathways, metabolism of cofactors and vitamins, and so on. The remaining 15 pathways were related to cellular processes, organismal systems, environmental information processing, and unclassified, respectively.

**Table 2:** Functional capacity of the microbial communities associated with ginger intervention.

Pathway level 1	Pathway level 2	Pathway level 3	*p*
Human diseases	Cardiovascular diseases	Viral myocarditis	0.00147
		Hypertrophic cardiomyopathy (HCM)	0.00258
	Immune system diseases	Primary immunodeficiency	0.01610
	Infectious diseases	Tuberculosis	0.00015
		Toxoplasmosis	0.00144
		Influenza A	0.00147
		Chagas disease (American trypanosomiasis)	0.00806
		*Staphylococcus aureus* infection	0.01701
		Bacterial invasion of epithelial cells	0.02443
	Cancers	Colorectal cancer	0.00144
		Small cell lung cancer	0.00144
		Pathways in cancer	0.00529
Metabolism	Lipid metabolism	Ether lipid metabolism	0.01675
	Metabolism of other amino acids	Selenocompound metabolism	0.04825
	Metabolism of cofactors and vitamins	Nicotinate and nicotinamide metabolism	0.00171
		Retinol metabolism	0.0145
		Folate biosynthesis	0.03369
	Biosynthesis of other secondary metabolites	Caffeine metabolism	0.01655
		Flavonoid biosynthesis	0.02815
		Indole alkaloid biosynthesis	0.03283
	Energy metabolism	Photosynthesis proteins	0.0149
		Oxidative phosphorylation	0.03894
		Photosynthesis	0.0246
	Xenobiotics biodegradation and metabolism	Atrazine degradation	0.01183
		DDT* degradation	0.03116
		Bisphenol degradation	0.04208
Cellular processes	Cell growth and death	Cell cycle – Caulobacter	1.80E-05
		Apoptosis	0.00164
		p53 signaling pathway	0.00261
Organismal systems	Environmental adaptation	Plant-pathogen interaction	0.02162
		Circadian rhythm – plant	0.02634
	Digestive system	Protein digestion and absorption	0.02917
	Endocrine system	Renin-angiotensin system	0.00419
Environmental information processing	Signal transduction	MAPK signaling pathway – yeast	0.00997
	Membrane transport	ABC transporters	0.00658
		Transporters	0.03684
Unclassified	Metabolism	Biosynthesis and biodegradation of secondary metabolites	0.00096
		Amino acid metabolism	0.04224
		Nucleotide metabolism	0.04976
	Cellular processes and signaling	Electron transfer carriers	0.00139
		Cell division	0.00214

## Discussion

This study provided the first evidence, to our knowledge, of the modulations of ginger juice supplementation on human gut microbiota. In this study, we did not identify any significant changes in appetite, the serum levels of triglyceride, cholesterol, HDL-C, LDL-C, or other physiological phenotypes in subjects after ginger juice intervention (not shown). These results were possibly due to the strong ability to maintain homeostasis in healthy people (Kang et al., 2016), the less sufficient dose and duration of ginger interventions, and the eating habits as usual during the whole study. Ginger supplementation modulated the gut microbiota composition, however, and the relative abundance of microbial taxa, whereas bacterial diversity both in community evenness and in richness varied, which was consistent with previous reports (Wang et al., 2017, 2019).

In this controlled intervention trial, we found that short-term supplementation of ginger juice increased the Firmicutes-to-Bacteroidetes ratio and Proteobacteria abundance in healthy Chinese adults. These results were consistent with some but not all of the conclusions made in previous studies. As the two dominant bacterial phyla in the gut, Firmicutes and Bacteroidetes play important roles in the regulation of host lipid, bile acid, and sugar metabolism (Tremaroli and Backhed, 2012). The change of the Firmicutes-to-Bacteroidetes ratio is closely related to obesity (Anhe et al., 2018), with an increase in the relative abundance of Firmicutes and a decrease in the relative abundance of Bacteroides observed in both diet-induced and genetically obese mouse models as well as in humans (Ridaura et al., 2013; Chang et al., 2015). Study of Zhang et al. (2019) demonstrated that daily supplementation with fresh Angelica keiskei juice alleviated high-fat diet-induced obesity in mice by modulating gut microbiota composition, including a decrease in the Firmicutes-to-Bacteroidetes ratio. Obviously, our study did not produce such a change, but it showed opposite results for the Firmicutesto-Bacteroidetes ratio. Interestingly, a randomized, double-blind, placebo-controlled study demonstrated a minor beneficial effect of ginger supplementation (2 g/day ginger powder) for 12 weeks on weight loss and some metabolic features of obesity (Alizadeh-Navaei et al., 2008). Overall, ginger consumption appeared to have the potential to manage obesity. Wang et al. (2019) found ginger powder had beneficial effects on the prevention of obesity through modulation of gut microbiota in mice. The abundance of Proteobacteria in the normal chow diet-fed mice was notably higher after ginger supplementation, which was in agreement with our results. In their research, however, no significant changes were found in the level of the Firmicutes-to-Bacteroidetes ratio, possibly because of the intrinsic similarities and differences that exist between the human and murine core gut microbiota (Nguyen et al., 2015). Specifically, ginger juice interventions increased the Firmicutes-to-Bacteroidetes ratio, which also has been observed in populations with short-term dietary capsaicin or whole-grain intervention and in healthy subjects after the long-term consumption of vegetables, dietary fibers, and whole grain (Wu et al., 2011; Kang et al., 2016).

The gut microbiota composition and dysbiosis influence on the hormone orchestration indirectly affected appetite (Mitev and Taleski, 2019). Leptin is a hormone produced mainly by adipose tissue that inhibits appetite and fat synthesis and increases energy consumption to control body weight when present at higher levels. It has been suggested that a significant negative correlation exists between the plasmatic levels of leptin and the genus *Prevotella* (Mitev and Taleski, 2019; Pushpanathan et al., 2019). In this study, we found that the ginger group presented a lower *Prevotella* genus level (6.22%) compared with the control group (12.5%) at time point T7 (Supplementary Table S1). Moreover, although the ginger group presented a higher *Bacteroidetes* genus level (13.93%) compared with the control group (12.66%), ginger juice interventions decreased the Prevotella-to-Bacteroides ratio. Another study showed that the Prevotella-to-Bacteroides ratio was positively correlated with the total plasma cholesterol levels (Roager et al., 2014).

In addition, we found that pro-inflammatory (Hall et al., 2017) *Ruminococcus_1* went from 1.5% of total microbial abundance to 0.9%, the abundance of genus *Ruminococcus_2* decreased from 2.1 to 1.79%, and the anti-inflammatory butyrate producer (Hills et al., 2019) *Faecalibacterium* experienced an expansion of 5.85–7.79% microbial abundance (Supplementary Table S1). It has been reported that a decrease in *Ruminococcus*, a member of the Clostridia class responsible for degrading resistant starch, also closed relative to an increase in butyrate production (Valles-Colomer et al., 2019). These results indicated that the anti-inflammatory effects of ginger may be at least partially due to variations in the relative abundance of these butyrate-related species. Collectively, these results showed that short-term intake of ginger juice had substantial effects on the composition and function of gut microbiota in healthy people.

In the present study, PICRUSt analysis and additional metagenomic-predicted studies showed that the relative abundance of microbial genes was associated primarily with human disease-related pathways, including cardiovascular diseases, immune system diseases, infectious diseases and cancers, and metabolism pathways, including lipid metabolism, amino acid metabolism, and energy metabolism. Most of these findings were consistent with ginger’s rich pharmacological effects and potential to treat therapeutic diseases.

Moreover, we observed that daily consumption of ginger juices differentially affected the gut microbiota profile of male and female subjects. This difference was reflected mainly in the α-diversity of gut microbiota after ginger intervention. We observed significant changes in community evenness (Shannon’s index) for men and significant changes in richness (Chao1) for women. When we did not consider the gender factor, bacterial diversity both in community evenness and in richness caused by ginger intervention showed differences. We also analyzed the species with significant differences after ginger intervention in male and female subjects, which revealed that there were unique and shared species with significant differences in the male and female subjects following ginger intervention. Bolnick et al. (2014) demonstrated that the diet-microbiota association was sex dependent. Dietary Daikenchuto, which is a herbal medication, composed of ginger, ginseng, and Japanese pepper, is widely used in Japanese traditional Kampo medicine. One study found that Daikenchuto changed mouse microbiota differently in female and male animals (Miyoshi et al., 2018). In our study, we speculated that this sex difference might occur as a result of differences in the composition of microbiota between sexes at the start of ginger intervention and to a difference in the metabolism and bioactive-conversion of ginger in the two sexes. Although, we found significantly different phyla and genera between the control and ginger groups in men and in women, respectively. Unfortunately, most of the species that had significant differences following ginger intervention showed low abundance, regardless of men or women. Because of the lack of research on such intestinal bacteria of low abundance, the biological and clinical significance of many differentially expressed gut microbiota remains unclear. Even so, our findings underscored the importance of analyzing both male and female individuals to investigate the effects of ginger on gut microbiota. Additional studies are necessary to confirm these findings.

## Data Availability Statement

The original contributions presented in the study are publicly available. This data can be found at: http://www.biosino.org/node/project/detail/OEP001074.

## Ethics Statement

The studies involving human participants were reviewed and approved by Ethics Committee of Affiliated Hospital of Shandong University of Traditional Chinese Medicine. The patients/participants provided their written informed consent to participate in this study. Written informed consent was obtained from the individual(s) for the publication of any potentially identifiable images or data included in this article.

## Author Contributions

The authors’ responsibilities were as follows: SW and YL conceived and designed the overall research and interpreted data. DQ, DZ, and ShuZ were responsible for recruitment. The clinical aspects of the study were supervised by YL. ShaZ, HJ and XP performed the measure of blood biochemical items. SG, HZ, QX, and LC performed the preparation of ginger juice and reception of fecal samples from the subjects. XW analyzed and interpreted data and wrote the paper. DQ edited the paper. All authors contributed to the article and approved the submitted version.

### Conflict of Interest

The authors declare that the research was conducted in the absence of any commercial or financial relationships that could be construed as a potential conflict of interest.

## Footnotes

^1^https://docs.qiime2.org/2019.1/

^2^http://picrust.github.com/
