# Supplementary material for: Gut Microbiota Variation With Short-Term Intake of Ginger Juice on Human Health
Source: Front Microbiol. 2021 Feb 23;11:576061. doi: 10.3389/fmicb.2020.576061 (PMC7940200; doi:10.3389/fmicb.2020.576061)
Supplement: Supplementary file 1 [file Table_1.pdf]

Supplemental Table 1. Community analysis on genus level at time point T7

Bacteria	Ginger_T7<br>Perentage(%)	Control_T7<br>Perentage(%)
Blautia	--	1.18
[Ruminococcus]_torques_group	0.93	1.14
Ruminococcus_1	0.90	1.50
Thauera	2.52	--
Acinetobacter	--	2.02
Subdoligranulum	1.31	1.67
Ruminococcaceae_UCG-002	1.77	1.38
Ruminococcaceae_UCG-014	2.18	--
Pseudomonas	2.38	1.19
Phascolarctobacterium	2.18	1.54
Ruminococcus_2	1.79	2.10
Megamonas	1.67	2.46
Lachnoclostridium	2.03	2.19
Bifidobacterium	1.99	2.37
Brevundimonas	1.93	5.28
Roseburia	3.19	4.55
Dialister	4.43	3.62
Faecalibacterium	7.79	5.85
[Eubacterium]_coprostanoligenes_group	9.30	5.40
[Eubacterium]_rectale_group	6.19	8.95
Prevotella	6.22	12.50
Bacteroides	13.93	12.66
Others	23.90	19.26
